# Challenges in the diagnosis of primary squamous cell carcinoma of the prostate: a case report and literature review

**DOI:** 10.3389/fsurg.2025.1532669

**Published:** 2025-07-17

**Authors:** Shengyou Song, Yalin Song

**Affiliations:** Department of Urology, Zaozhuang Municipal Hospital, Zaozhuang, China

**Keywords:** primary prostate cancer, squamous cell carcinoma, potential cancer, PSA, case report

## Abstract

**Introduction:**

Prostate squamous cell carcinoma (SCCP)is a rare malignancy that accounts for 1% of prostate cancer cases. In resource-limited settings, it is often at an advanced stage due to the limitations of PSA/imaging-based methods, and pathological confirmation is needed for a definitive diagnosis, particularly in elderly patients with comorbidities.

**Case Presentation:**

A 71-year-old male with benign prostatic hyperplasia presented with urinary obstruction confirmed by urine flowmetry. Digital rectal examination of the prostate revealed severe enlargement, a firm consistency and an irregular surface; B-mode ultrasonography revealed calcifications without focal lesions. Laboratory tests revealed hematuria, elevated RBC counts, reduced WBC counts, normal serum PSA, and negative microbiological cultures. Cystoscopy revealed bladder wall thickening with multiple diverticula, suggesting chronic obstructive sequelae. Through physical, laboratory, and imaging examinations, we diagnosed the patient with benign prostatic hyperplasia before surgery. The postoperative pathological diagnosis was SCCP. The patient was discharged 7 days post-surgery and treated for prostate cancer (PCa) at a higher-level hospital.

**Conclusion:**

*in vivo* fluorescence imaging and laboratory examination of PCa targets are needed to further promote noninvasive PCa diagnosis.

## 1 Introduction

Prostate cancer (PCa) is the second most common malignancy (after lung cancer) in men worldwide, and its morbidity and mortality rates increase with age (1). Squamous cell carcinoma (SCC) of the prostate (SCCP) is a rare disease that accounts for approximately 1% of all PCa cases, and most PCa patients are diagnosed at an advanced stage. At this stage, the survival time is shorter and fewer treatment options are available (2).

At present, the early diagnosis of SCCP and other histological types of PCa requires serum prostate-specific antigen (PSA) measurement and imaging examination. Imaging-based fluorescence localization (IFL) and laboratory immunotargeting techniques sometimes cannot be implemented and their effectiveness is limited due to medical constraints (the restrictions of patient funding and hospital testing equipment). However, when the two examinations are negative or if the result indicates prostatic hyperplasia, it is difficult to determine whether pathological biopsy is needed, especially for elderly patients who are in poor physical condition, show relevant symptoms of urinary tract obstruction, or require urgent surgical intervention. This situation also makes diagnosis difficult, and this type of potential primary PCa can be identified only through pathological examination.

This paper reports the discovery and characterization of a case of primary SCCP. We hope that this report will help clinicians develop better diagnostic and treatment methods for malignant tumors associated with a poor prognosis. We present this case series in accordance with the AME Case Series reporting checklist*.

## 2 Case presentation

The patient was a 71-year-old man who was admitted to the hospital on May 29, 2021, with a chief complaint of urinary frequency and urgency, painful urination combined with difficulty urinating for half a year, and hematuria for 3 days. The frequent urination and difficulty urinating that had begun six months prior had no obvious causes, and the patient had not sought systematic treatment.

**Figure 1:**
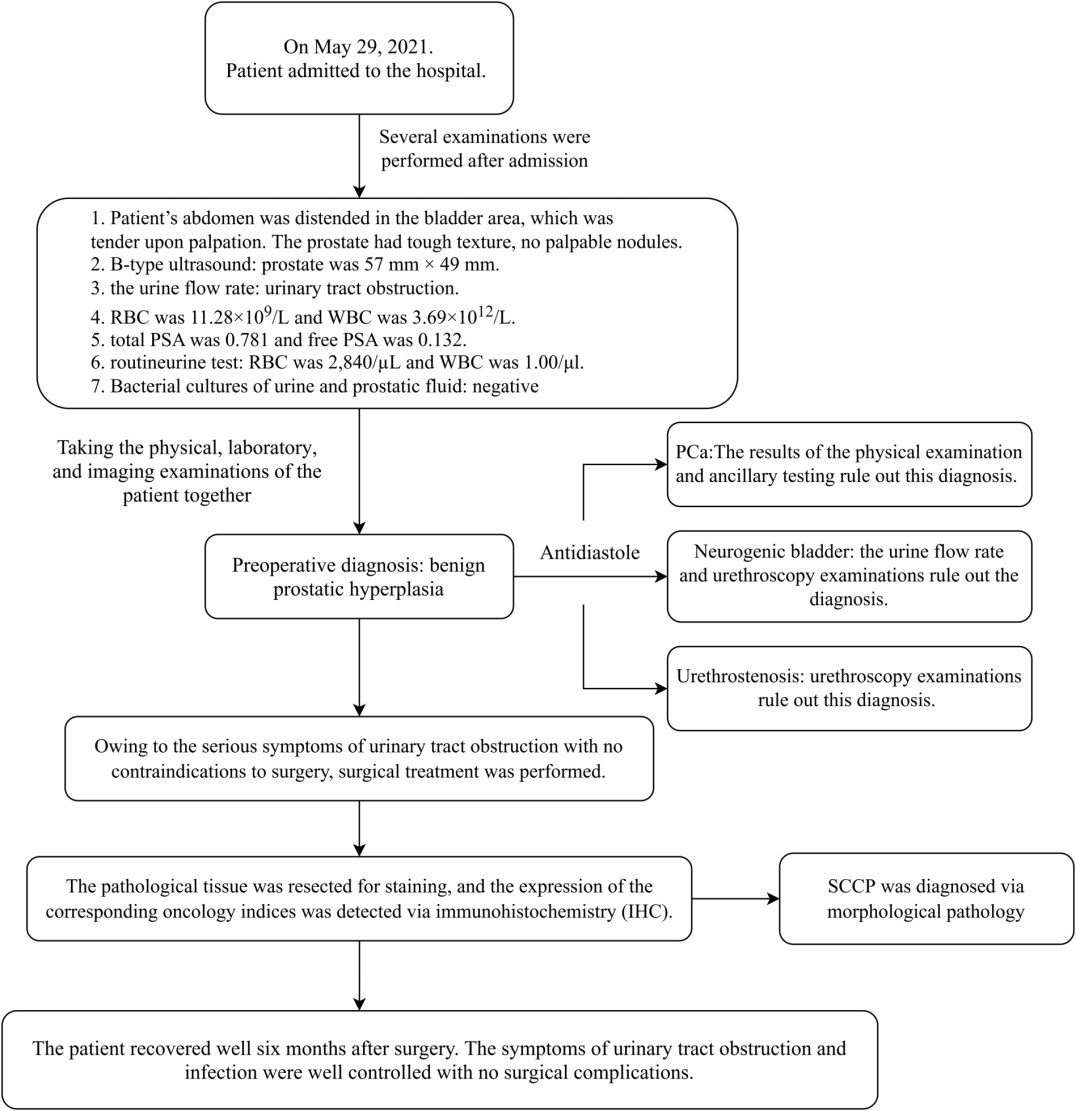
Timeline of patient care. The patient was seen and admitted for diagnosis and treatment process and was ultimately discharged to receive treatment at a higher-level hospital.

After admission, relevant examinations revealed that the patient's abdomen was distended in the bladder area, which was tender upon palpation. The digital rectal examination (DRE) revealed that the prostate demonstrated Grade III benign prostatic hyperplasia (transverse diameter >5 cm) with a firm consistency and irregular surface contour. No palpable focal nodules or space-occupying lesions were detected. The anal sphincter tone was within normal limits. The rectal mucosa appeared smooth without evidence of extrinsic compression. No blood staining was observed on the gloved finger upon withdrawal. Imaging examination via B-type ultrasound revealed that the patient's prostate was 57 mm × 49 mm (reference interval, 40 mm × 30 mm), with a shallow central sulcus and calcification of the prostate. Examination of the urine flow rate suggested urinary tract obstruction. Routine blood tests revealed that the patient's red blood cell (RBC) count was 11.28 × 10^9^/L (reference interval, 3.5–9.5 × 10^9^/L), his white blood cell (WBC) count was 3.69 × 10^12^/L (reference interval, 4.3–5.8 × 10^12^/L), his total serum PSA level was 0.781 (reference interval, 0–4 ng/ml), and his free PSA level was 0.132 (reference interval, 0–0.42 ng/ml). A routine urine test suggested hematuria, with an RBC count of 2,840/µl (reference interval, 0–5/µl) and a WBC count of 1.00/μl (reference interval, 0–9/µl). Bacterial cultures of urine and prostatic fluid were negative. Cystoscopy revealed thickening of the bladder wall and the presence of multiple diverticula. Through physical, laboratory, and imaging examinations of the patient together, a preoperative diagnosis of benign prostatic hyperplasia was made. Differential diagnosis to exclude other diseases included physical examination and auxiliary examination. The diagnostic details are shown in Figure 1. The patient denied a similar family history and genetic history.

**Figure 2:**
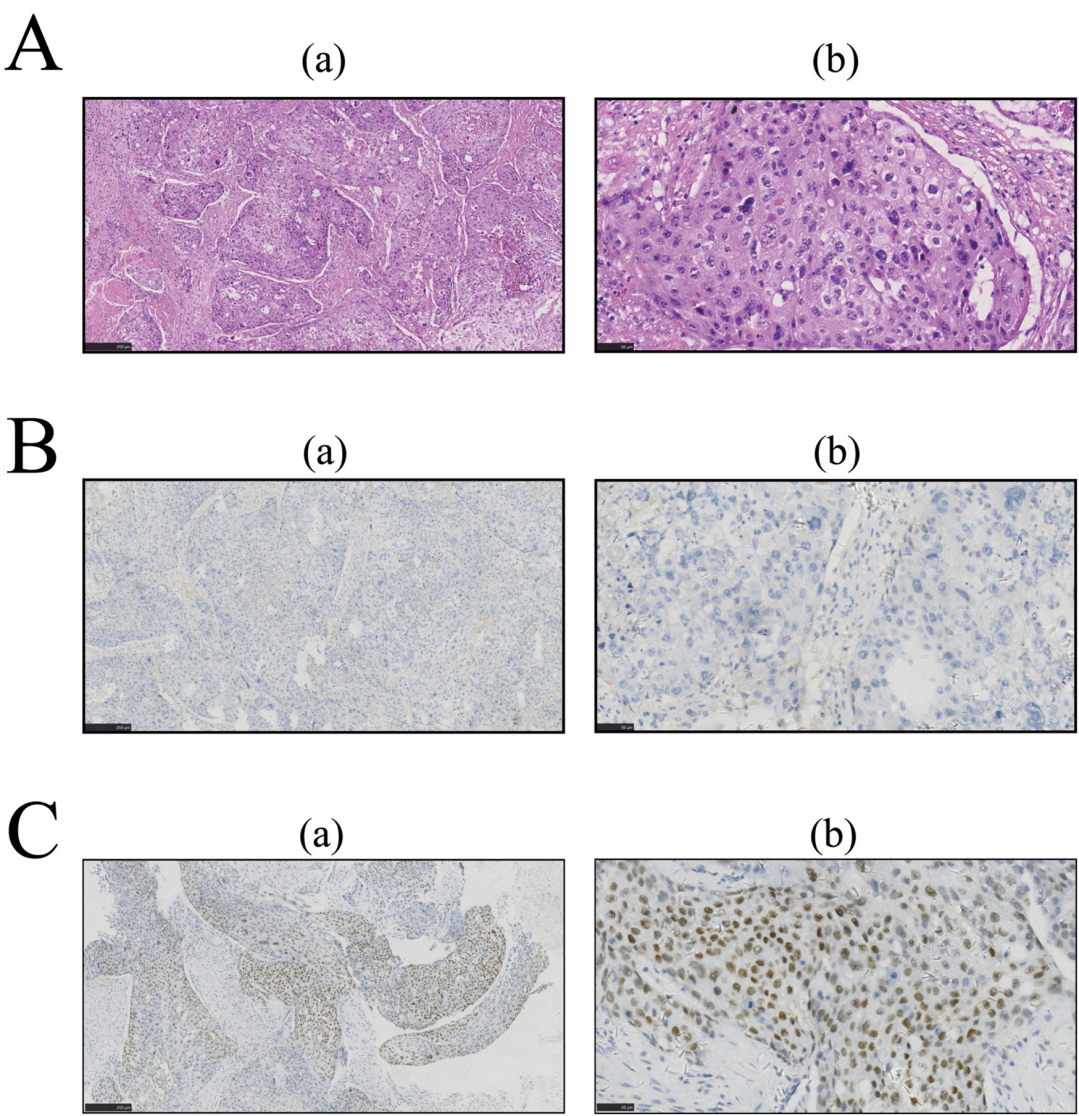
After paraffin embedding, prostate tissue sections were subjected to immunohistochemistry (IHC) and hematoxylin and eosin (HE) staining. The images in column **(a)** were taken at low magnification (5×); the images in column **(b)** were taken at high magnification (20×). Pathological examination of resected prostate tissue by HE. IHC was performed for further examination, and the results were analyzed with ImageJ software. **(A)** HE staining. **(B)** IHC for PSA. **(C)** IHC for GATA-3.

**Figure 3:**
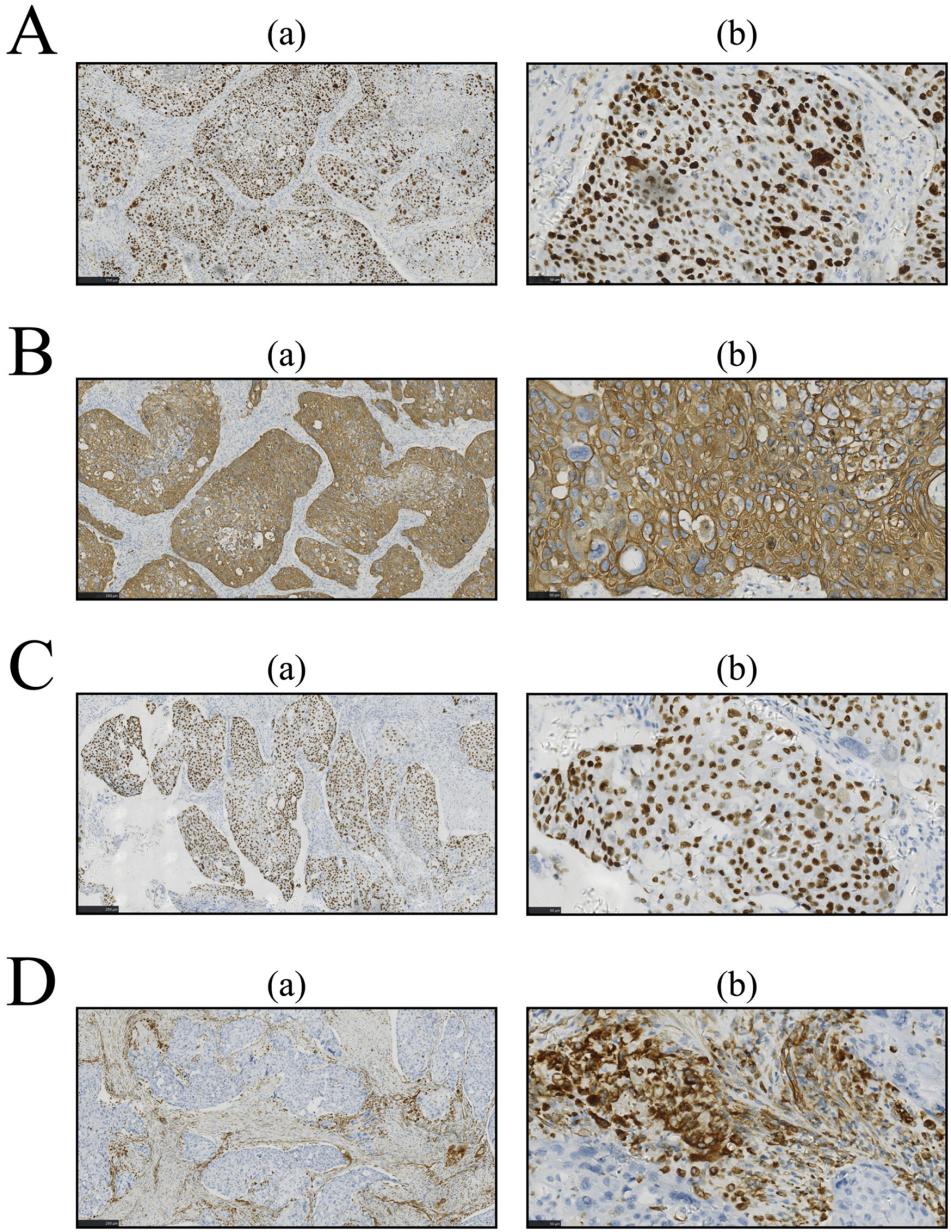
Immunohistochemistry (IHC) was performed for further examination, and the results were analyzed with imageJ software. Sections were prepared as described in Figure 3. **(A)** Ki-67 staining by IHC. **(B)** IHC for CK5/6. **(C)** IHC for p63. **(D)** IHC for vimentin.

Owing to the serious symptoms of urinary tract obstruction, medical intervention is urgently needed. After confirming that there were no contraindications to surgery, surgical treatment was performed. The pathological tissue was resected for staining, and the corresponding oncological indices were detected via immunohistochemistry (IHC). The results are shown in Figures 2, 3, respectively. The pathological morphology of the tumor was observed via hematoxylin and eosin (HE) staining, the SCCP was diagnosed via morphological pathology and the pathological stage was pT1bN0M0I. Pathological examination of the resected prostate tissue via HE staining revealed moderately to poorly differentiated SCC (Figure 2A), with cancer cells gathered in a cancer nest. The tumor was wrapped with pericancerous tissue, which contained a few cancer cells and blood vessels. The IHC results were negative for PSA, focally positive for GATA binding protein 3 (GATA-3), and positive for cytokeratin 5/6 (CK5/6), Ki-67, p63, and vimentin. Furthermore, CK5/6 was expressed mainly in the cytoplasm of cancer cells in the nest (Figure 3B). GATA-3 (Figure 2A), Ki-67 (Figure 3A) and p63 (Figure 3C) were expressed mainly in the nucleus, and vimentin (Figure 3D) and CK5/6 (Figure 3B) were expressed mainly in the pericancerous tissue. The HE and IHC results indicated that the tumor was somewhat aggressive. While treating this patient's prostate disease, a differential diagnosis of prostate cancer, neurogenic bladder, or urethral stricture was considered (Figure 1). However, on the basis of laboratory and imaging findings, benign prostatic hyperplasia with urinary tract infection was more likely. Second, symptoms of urinary tract obstruction are serious and require urgent surgical intervention to remove the obstruction, unless there are contraindications to surgery. After surgical treatment, pathological examination of the tissue revealed that the patient had started to develop primary moderately to poorly differentiated squamous cell carcinoma in the prostate tissue; this case also partly reflects the present situation of prostate cancer diagnosis and treatment. Therefore, further exploration of the diagnostic and examination methods for primary squamous cell carcinoma of prostate cancer is needed.

Postoperative follow-up at six months revealed favorable clinical outcomes. The patient exhibited complete resolution of the lower urinary tract obstruction and associated infectious sequelae, with successful catheter removal achieved seven days after the procedure. Urodynamic evaluation revealed marked improvement in voiding efficiency, as evidenced by a 7.6-fold increase in the maximum urinary flow rate (Qmax) from 3.3 ml/s preoperatively to 25 ml/s post-intervention. These quantitative metrics meet the established criteria for successful bladder outlet obstruction management (Schäfer nomogram Grade 0), confirming definitive resolution of voiding dysfunction (3). The absence of perioperative complications (Clavien‒Dindo Grade 0) and sustained therapeutic efficacy align with contemporary outcomes for minimally invasive prostatic surgery. Radical prostatectomy was not performed because of the patient's age. The patient was discharged from the hospital 7 days after surgery, and treatment for PCa was initiated at a higher-level hospital. Following disclosure of the postoperative histopathological findings through proactive clinician‒patient counseling, the patient demonstrated full comprehension and acceptance of the diagnostic results. Notably, the patient expressed commitment to strict adherence to the proposed therapeutic regimen and active participation in scheduled follow-up surveillance protocols. The timeline of the patient's treatment course is shown in Figure 1.

## 3 Discussion

PCa is a prevalent malignancy in elderly men and the second leading cause of cancer-related death in the US (4); notably, SCCP accounts for <1% of prostate malignancies (5). Unlike adenocarcinoma, SCCP lacks PSA secretion and androgen receptor dependence, making preoperative diagnosis exceptionally challenging (6). Early diagnosis is crucial for improving patient survival. Screening methods include serum PSA measurement and magnetic resonance imaging (MRI); however, MRI can be expensive, and the role of serum PSA in cancer screening is controversial (7).

This case met three of the core diagnostic criteria of Mott et al. (5): examination of: continuous sections confirmed that the tumor's squamous differentiation area reached 95% (Figure 2A); whole-body PET-CT (imaging number: A016DX2206010175) excluded metastatic squamous carcinoma; and serum and prostate-specific antigen (PSA) were both negative in the cancer tissue (Figure 2B). Immunohistochemical analysis revealed that the basal cell marker p63 was strongly positive in the nucleus (Figure 3C), while the characteristic adenocarcinoma marker p504s was negative which is consistent with the squamous carcinoma-specific diagnostic model proposed by Dizman et al. (6). The mechanism of PCa formation remains unknown, but it could be due to gene mutations or amplification of the androgen receptor (8). Notably, with respect to the tumor origin and tumorigenesis mechanism: (1) although no adenocarcinoma component was found, the lack of ERG gene break detection (FISH verification) made it difficult to completely rule out the adenocarcinoma dedifferentiation hypothesis (9); moreover, TMPRSS2-ERG gene fusions and SPOP alterations can be detected during PCa tumorigenesis and invasion (10); (2) although CD44 membrane expression was not observed, the coexpression of p63 (basal marker) and vimentin (stromal marker) around the cancer nest (Figures 3C,D) aligned with the theoretical model in which tumor stem cells drive metastasis through epithelial‒mesenchymal transition (EMT) (11–13), supporting the pluripotent stem cell origin hypothesis; and (3) the lack of a history of radiotherapy enabled exclusion of the treatment-induced transformation mechanism (14).

Despite their noninvasive nature and advantages of painlessness, convenience, and rapid results, diagnostic imaging modalities demonstrate lower sensitivity in detecting primary SCCP than do pathological examinations. Similar to our reported case, SCCP typically presents with overlapping clinical manifestations (e.g., dysuria and urinary frequency) and imaging features (such as extensive prostatic lesions on multiparametric MRI) with prostatic adenocarcinoma (15). However, the PSA level in SCCP generally remains within the normal range or is only mildly elevated (16–18). This characteristic of PSA-insensitivity frequently contributes to diagnostic errors or delayed diagnosis, as evidenced by reported cases with PSA levels as low as 1.62 ng/ml (15, 19), which are significantly below typical adenocarcinoma thresholds. While FDG-PET may reveal hypermetabolic foci in both primary prostatic lesions and metastatic lymph nodes (15), such findings lack specificity for squamous differentiation as they are also observable in locally advanced or metastatic adenocarcinomas (18, 19). Notably, osseous metastases may manifest as lytic lesions, which contrasts with the osteoblastic pattern typical of adenocarcinomas, so pathological confirmation is needed (17). The current consensus maintains that conventional imaging techniques play primarily adjunctive roles in SCCP diagnosis, with a definitive diagnosis ultimately requiring histopathological verification. The outlined challenges in prostatic squamous cell carcinoma (SCCP) imaging-based diagnosis are critically exemplified in this case. Histopathological evaluation confirmed the aggressive biological behavior of the primary SCCP, which indicated a poor prognosis. Immunohistochemical analysis revealed the absence of PSA expression in both the serum and neoplastic tissues (Figure 2B). This biochemical profile likely reflects two biological mechanisms: (1) tumor biological behavior characterized by an active proliferative phase with suppressed secretory differentiation, and (2) the intrinsic nonsecretory phenotype of SCC histology, which fundamentally differs from that of conventional PCa in maintaining the PSA-negative status throughout carcinogenesis. GATA-3 (Figure 2C) was focally expressed in the SCCP tissue of this patient, and Ki-67 and p63 were concentrated in the nucleus of cancer cells in the cancer nest. GATA-3 is an important prognostic marker for urological cancer. Because GATA-3 and Ki-67 (20) are crucial prognostic markers for urological cancer diagnosis, high expression often indicates poor cancer differentiation and prognosis (21). Additionally, the role of p63 in tumor development remains controversial. Several studies (22) have shown that by inducing p63-mediated G1/M cell cycle arrest, cancer cell proliferation can be inhibited. Some studies have shown that amplification or deletion of the p63 locus disrupts the cellular microenvironment and leads to tumorigenesis (21). We speculated that the p63 gene and its family of proteins might exert antitumor effects by inhibiting tumor growth and tumor phenotypes. The marked upregulation of p63 may explain why this patient did not exhibit obvious indications of a tumor. Another study showed (23) that the vimentin protein is involved in the development of the invasive phenotype of tumor tissues and is expressed mainly in poorly differentiated PCa and bone metastasis tissues, whereas it is almost undetectable in well-differentiated or moderately differentiated PCa. Because vimentin, which is involved in tumor invasiveness (23), was expressed mainly in the pericancerous SCC tissue of this patient, an invasive phenotype of cancer tissue could not be excluded. CK5/6 is currently an important marker for the diagnosis and prognosis of breast cancer and for tumor differentiation, and its positive expression often predicts poor patient prognosis (24). In the patient's tumor tissue, the IHC results revealed strong positive expression of CK5/6 (Figure 3B) in the patient, which was concentrated in the cancer cell mass in the cancer nest, suggesting that the primary prostate squamous cell carcinoma in this case may still be highly aggressive with a poor prognosis.

**Figure 4:**
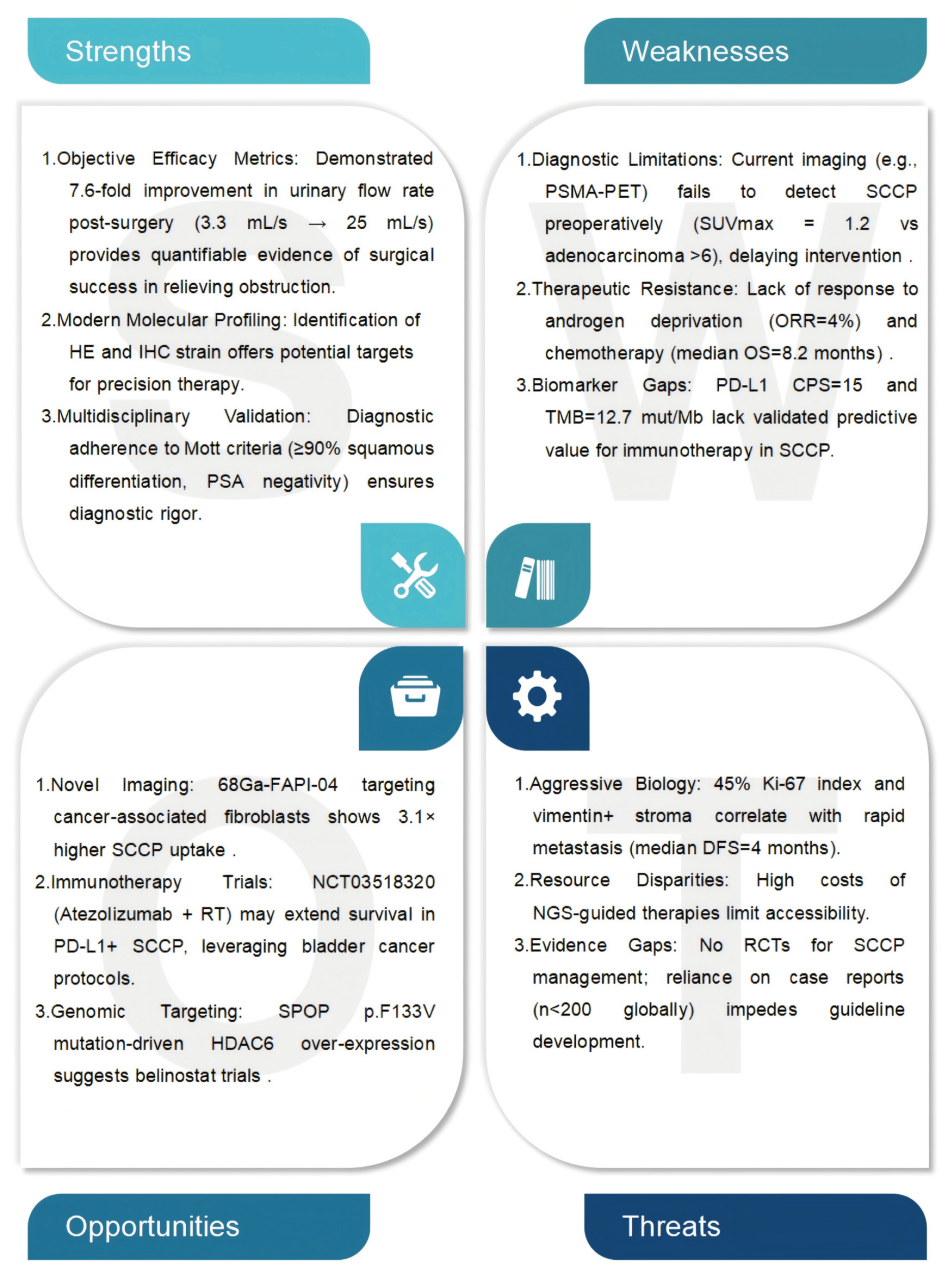
SWOT analysis of squamous cell carcinoma of the prostate (SCCP). Clinical observations and therapeutic outcomes from the case study were used to further support the diagnosis of SCCP.

While current diagnostic modalities enabled SCCP identification and risk stratification in this patient, broader implementation of noninvasive technologies may significantly increase diagnostic sensitivity, facilitating early interventions to reduce SCCP-related mortality. Advanced strategies such as fluorescence-guided localization and immune-targeted therapeutics hold particular promise for overcoming these limitations. The fluorescence localization technology employs molecule-specific fluorescent probes conjugated with target proteins or ligands (e.g., IRDye800CW-labeled Fc-domain proteins) to achieve real-time, noninvasive visualization of tumor-associated immune cell infiltration, thereby revolutionizing immunophenotyping and treatment monitoring (25). Recent progress in precision localization technology for subcellular targeting has been exemplified by innovations in *Eimeria* vaccine development, where the fusion of enhanced yellow fluorescent protein (EYFP) with surface antigens (SAG1) or microneme proteins (MIC2) enables antigen localization to sporozoite surfaces or secretory organelles, optimizing vaccine immunogenicity (26). Immune-targeting technology leverages engineered cellular therapies and delivery systems to achieve tissue-specific biodistribution. For example, chemokine receptor-modified T-cells (e.g., CCR4- and CCR6-engineered variants) selectively home to tumor-draining lymph nodes and intratumoral regions in murine colon cancer models, markedly improving therapeutic efficacy (27). Near-infrared photoimmunotherapy (NIR-PIT) further combines antibody-mediated targeting (e.g., anti-EGFR) with phototoxic activation to induce immunogenic cell death while stimulating systemic antitumor immunity (28). The integration of fluorescence localization technology and immune targeting technology has catalyzed the development of multifunctional nanoparticle platforms. The DIANA system utilizes passive targeting (intravenous micelles) or cotransplantation strategies (nanofiber scaffolds) to deliver fluorescent-tagged immunomodulators to inflammatory or graft sites, achieving localized immune regulation with minimal systemic toxicity (29). Similarly, complementary studies employing fluorescence-tracked IL-15/anti-PD-1 combinations have revealed dynamic spatiotemporal interactions between tumor microenvironments and secondary lymphoid organs, revealing mechanistic synergies in immunotherapy (30). Despite these advancements, technological limitations and regional healthcare disparities precluded their application in the management of this case of SCCP. These findings underscore the urgent need for multicenter validation of fluorescence-based biomarkers, development of cost-effective delivery systems, and establishment of policy initiatives to address global healthcare imbalances. Accelerating the clinical translation of these precision technologies may improve SCCP management by facilitating earlier diagnosis and personalized treatment and thus may improve survival outcomes (Figure 4).

SCCP and PCa treatment predominantly rely on surgical intervention. However, compared with PCa, SCCP exhibits distinct biological behavior and therapeutic responses, with metastatic SCCP remaining particularly refractory to existing treatments. The first key difference is that, SCCPs exhibit intrinsic resistance to hormonal therapies. While PCa progression typically depends on androgen receptor (AR) signaling, which makes androgen deprivation therapy (ADT) a cornerstone treatment, SCCP lacks AR expression and is not characterized by abnormal PSA levels; in fact, serum PSA levels often remain normal even during disease progression (17, 31). In a representative case, rapid metastatic dissemination was observed in a 48-year-old patient with adenosquamous carcinoma despite ADT administration (17). Second, conventional chemotherapy has demonstrated limited efficacy in SCCP. Although PCa patients exhibit partial responsiveness to docetaxel-based regimens, SCCP displays inherent resistance to platinum agents and taxanes. Clinical reports indicate that combination chemotherapy (e.g., carboplatin plus docetaxel) provides only transient disease control in adenosquamous variants, often necessitating subsequent radiotherapy or targeted therapies (32). This chemoresistance may stem from both the absence of actionable molecular targets in the SCCP tumor microenvironment and its aggressive progression kinetics, which preclude sustained therapeutic responses (31, 33). Furthermore, genomic analyses revealed profound molecular divergence between SCCP and adenocarcinoma. The tumor suppressor FBXW7, which is frequently downregulated in adenocarcinomas, is not expressed in SCCPs, and this phenotype leads to the accumulation of oncoproteins (e.g., c-MYC) that drive invasiveness (34). The squamous differentiation marker ΔNp63α enhances metastatic potential through CD82-mediated modulation of bone microenvironment adhesion and cancer stemness (35). Additionally, heterogeneous prostate-specific membrane antigen (PSMA) expression in SCCP metastases compromises the efficacy of PSMA-targeted radioligand therapies such as 177Lu-PSMA (36, 37). These molecular aberrations — encompassing hormonal independence, chemoresistance, and unique genomic signatures — collectively explain the lack of standardized therapies for metastatic SCCP. Notably, multiomics profiling and innovative targeted/immunotherapeutic strategies need to be employed to facilitate new therapeutic breakthroughs.

The breadth of minimally invasive treatments for prostate cancer, have significantly increase; these treatments include high-intensity focused ultrasound (HIFU), laparoscopic/robotic-assisted prostatectomy, cryotherapy, photodynamic therapy, and brachytherapy. Among these, HIFU has shown considerable precision in localized disease management, achieving a median PSA nadir of 0.15 ng/ml and a negative biopsy rate of 66.1% at 6 months, with optimal outcomes when the baseline PSA is <10 ng/ml and the nadir is <0.40 ng/ml (38, 39). Robotic-assisted laparoscopic prostatectomy (RALP), a minimally invasive surgical approach, offers comparable oncological control to open radical prostatectomy while reducing blood loss (median 200 ml vs. 900 ml) and hospital stay (1–2 days vs. 3–5 days) (40, 41). For radiation-recurrent tumors, salvage HIFU and cryotherapy yield equivalent 5-year cancer-specific survival rates to salvage prostatectomy (78%–82% vs. 81%) but markedly lower urinary incontinence rates (15%–20% vs. 45%) (42, 43). Comparative studies suggest that the precision of HIFU in focal ablation surpasses that of cryotherapy (74% vs. 64% negative margins) but requires longer procedure times (2.5 vs. 1.8 hours) (39, 43). Emerging modalities such as vascular-targeted photodynamic therapy (VTP) have exhibited 73% complete ablation rates in index lesions while preserving sexual function in 85% of cases (43). However, microwave thermotherapy is only indicated for benign prostatic hyperplasia because of insufficient cancer-specific efficacy (44). Current guidelines recommend these techniques primarily for low-intermediate risk patients (Gleason ≤7, PSA ≤20 ng/ml) with prostate volumes <40 ml (41, 45). Long-term data remain scarce, with only 43% of HIFU studies reporting ≥5 years of follow-up (45, 46). Technical innovations such as real-time MRI-ultrasound fusion guidance (precision ± 1.2 mm) and temperature-controlled ablation zones are addressing historical limitations in tumor targeting (38, 41). Preliminary data suggest that these approaches can be extended to locally advanced disease if they are combined with androgen deprivation therapy, which results in a 68% 3-year biochemical-free survival rate in T3a patients (39). Generally, SCCP is considered more invasive than prostate adenocarcinoma (47). The patient's urine and blood test results, as well as imaging studies, all favored benign prostatic hyperplasia. Prostate infection, inflammation, or urinary stones involving the prostate can still lead to elevated serum PSA levels, whereas some patients with primary PCa exhibit negative AR and PSA results, obscuring clinicians’ judgment. Invasive prostate tissue biopsy is the most effective examination method. Although our treatment was appropriate for benign prostatic hyperplasia, pathological examination revealed the presence of SCCP. Our experimental results show that new treatment methods for PSA-negative PCa need to be explored. To identify specific targets in PCa fluorescence localization imaging and laboratory immune-targeting technology need to be implemented along with new noninvasive diagnosis and treatment methods for PCa, which may increase the PCa screening rate in elderly patients, reduce their surgical risk, alleviate the pain associated with invasive examinations and increase the feasibility of pathological examinations (Figure 4).

## 4 Conclusions

Primary PCa cannot be diagnosed noninvasively, as demonstrated in this case report. This disease is rare, particularly considering its histology. The majority of PCa patients are elderly males, and the specific targets of prostate cancer have been explored by modern means, however modern noninvasive examination methods still need to be applied to increase the accuracy of prostate cancer diagnosis for elderly patients.

## Data Availability

The datasets presented in this study can be found in online repositories. The names of the repository/repositories and accession number(s) can be found in the article/Supplementary Material.
